# BRCA1 positively regulates *FOXO3* expression by restricting *FOXO3* gene methylation and epigenetic silencing through targeting EZH2 in breast cancer

**DOI:** 10.1038/oncsis.2016.23

**Published:** 2016-04-04

**Authors:** C Gong, S Yao, A R Gomes, E P S Man, H J Lee, G Gong, S Chang, S-B Kim, K Fujino, S-W Kim, S K Park, J W Lee, M H Lee, KOHBRA study group, U S Khoo, E W-F Lam

**Affiliations:** 1 Department of Surgery and Cancer, Imperial College London, London, UK; 2 Li Ka Shing Faculty of Medicine, Department of Pathology, The University of Hong Kong, Hong Kong SAR, China; 3 Department of Pathology, Asan Medical Center, University of Ulsan College of Medicine, Seoul, Korea; 4 Department of Biomedical Sciences, Asan Medical Center, University of Ulsan College of Medicine, Seoul, Korea; 5 Department of Oncology, Asan Medical Center, University of Ulsan College of Medicine, Seoul, Korea; 6 Department of Surgery, Daerim St. Mary's Hospital, Seoul, Korea; 7 Department of Preventive Medicine, Seoul National University College of Medicine, Seoul, Korea; 8 Department of Surgery, Asan Medical Center, University of Ulsan College of Medicine, Seoul, Korea; 9 Department of Surgery, Soonchunhyang University Hospital, Soonchunhyang University College of Medicine, Seoul, Korea

## Abstract

BRCA1 mutation or depletion correlates with basal-like phenotype and poor prognosis in breast cancer but the underlying reason remains elusive. RNA and protein analysis of a panel of breast cancer cell lines revealed that BRCA1 deficiency is associated with downregulation of the expression of the pleiotropic tumour suppressor FOXO3. Knockdown of BRCA1 by small interfering RNA (siRNA) resulted in downregulation of FOXO3 expression in the BRCA1-competent MCF-7, whereas expression of BRCA1 restored FOXO3 expression in BRCA1-defective HCC70 and MDA-MB-468 cells, suggesting a role of BRCA1 in the control of FOXO3 expression. Treatment of HCC70 and MDA-MB-468 cells with either the DNA methylation inhibitor 5-aza-2'-deoxycitydine, the *N*-methyltransferase enhancer of zeste homologue 2 (EZH2) inhibitor GSK126 or EZH2 siRNA induced FOXO3 mRNA and protein expression, but had no effect on the BRCA1-competent MCF-7 cells. Chromatin immunoprecipitation (ChIP) analysis demonstrated that BRCA1, EZH2, DNMT1/3a/b and histone H3 lysine 27 trimethylation (H3K27me3) are recruited to the endogenous *FOXO3* promoter, further advocating that these proteins interact to modulate *FOXO3* methylation and expression. In addition, ChIP results also revealed that BRCA1 depletion promoted the recruitment of the DNA methyltransferases DNMT1/3a/3b and the enrichment of the EZH2-mediated transcriptional repressive epigenetic marks H3K27me3 on the *FOXO3* promoter. Methylated DNA immunoprecipitation assays also confirmed increased CpG methylation of the *FOXO3* gene on BRCA1 depletion. Analysis of the global gene methylation profiles of a cohort of 33 familial breast tumours revealed that *FOXO3* promoter methylation is significantly associated with BRCA1 mutation. Furthermore, immunohistochemistry further suggested that FOXO3 expression was significantly associated with BRCA1 status in EZH2-positive breast cancer. Consistently, high FOXO3 and EZH2 mRNA levels were significantly associated with good and poor prognosis in breast cancer, respectively. Together, these data suggest that BRCA1 can prevent and reverse FOXO3 suppression via inhibiting EZH2 and, consequently, its ability to recruit the transcriptional repressive H3K27me3 histone marks and the DNA methylases DNMT1/3a/3b, to induce DNA methylation and gene silencing on the *FOXO3* promoter.

## Introduction

Breast cancer is the most common type of cancer among women worldwide. BRCA1 is a multifunctional tumour suppressor, which has a key role in mammary tumorigenesis. BRCA1 dysregulation and/or mutation are closely associated with a higher risk of breast cancer in familial cases. Accordingly, reduced BRCA1 expression or mutation has been frequently reported in sporadic breast cancer.^1, 2^ BRCA1 silencing or mutation is also associated with basal-type breast cancer phenotype in which the tumour cells express no estrogen receptor (ER), progesterone receptor (PR) nor human epidermal growth factor receptor 2 (HER2) receptor, high p53 mutation and poor prognosis.^3^ The mechanisms linking BRCA1 loss/mutation to tumorigenesis are not clearly understood. Inactivation of BRCA1 has been shown to induce malignant cell behaviour including accelerated cell proliferation, enhanced anchorage-independent growth and suppressed expression of proliferative inhibitors p21^Waf1/Cip1^ and p27^Kip1^.^4^ In this context, BRCA1 can transcriptionally regulate p27^Kip1^ in cooperation with FOXA1.^5, 6^ BRCA1 also regulates expression of Survivin, an inhibitor of cell division and apoptosis,^7^ through regulating the expression of the NAD^+^-dependent deacetylase sirtuin-1.^8^ In addition, it has been reported that BRCA1 can negatively regulate phosphoinositide 3-kinase/AKT pathway by inducing AKT ubiquitination and degradation, resulting in FOXO3 hypophosphorylation and induction of FOXO3 activity.^9^

The transcription factor FOXO3 is a member of the Forkhead box (FOX) protein family and a well-established tumour suppressor, which transcriptionally regulates genes that are important in a myriad of cellular processes such as cell cycle progression,^10^ apoptosis,^11^ angiogenesis^12^ and chemotherapeutic drug response.^13^ High FOXO3 expression has been reported to be associated with low histological grades, low tumour stages, lymph node negativity and better survival in breast cancer patients,^14^ and has been shown to suppress the oestrogen-dependent breast cancer tumorigenesis *in vivo.*^15^ In addition, FOXO3 is also the central mediator of the pro-proliferative phosphoinositide 3-kinase/AKT signalling pathway in which AKT phosphorylation causes the inactivation, nuclear exclusion and subsequently degradation of FOXO3.^16^ Other kinases such as IκB kinase^17^ and extracellular signal-regulated kinase^18^ can also phosphorylate and downregulate FOXO3 activity in a similar manner, to promote cell proliferation and tumorigenesis.

EZH2 (enhancer of zeste homologue 2) is the enzymatic subunit of the polycomb-repressive complex 2 (PRC2) and a methyltransferase that specifically catalyses the addition of methyl groups to histone H3 at lysine 27 (H3K27). Trimethylation of H3K27 (H3K27me3) serves as an epigenetic code for further recruitment of other polycomb complexes, DNA methyltransferases (DNMTs) and histone deacetylases, leading to chromatin condensation and transcription suppression.^19^ EZH2 overexpression is associated with metastasis and poor survival in breast cancer patients.^20^ It has been shown that growth of *Brca1*-deficient mouse mammary tumours is dependent on EZH2 expression.^21^ Interestingly, BRCA1 can bind directly to EZH2 and negatively regulate its functions in both mouse embryonic stem cells and human breast cancer cells.^22^ In the current study, we sought to bridge BRCA1 and FOXO3, the two core tumour suppressors in breast cancer, focusing on the role of BRCA1 in regulating FOXO3 expression, and to delineate the regulatory mechanism involved.

## Results

### Correlation between BRCA1 and FOXO3 expression in breast cancer cell lines

**Figure 1:**
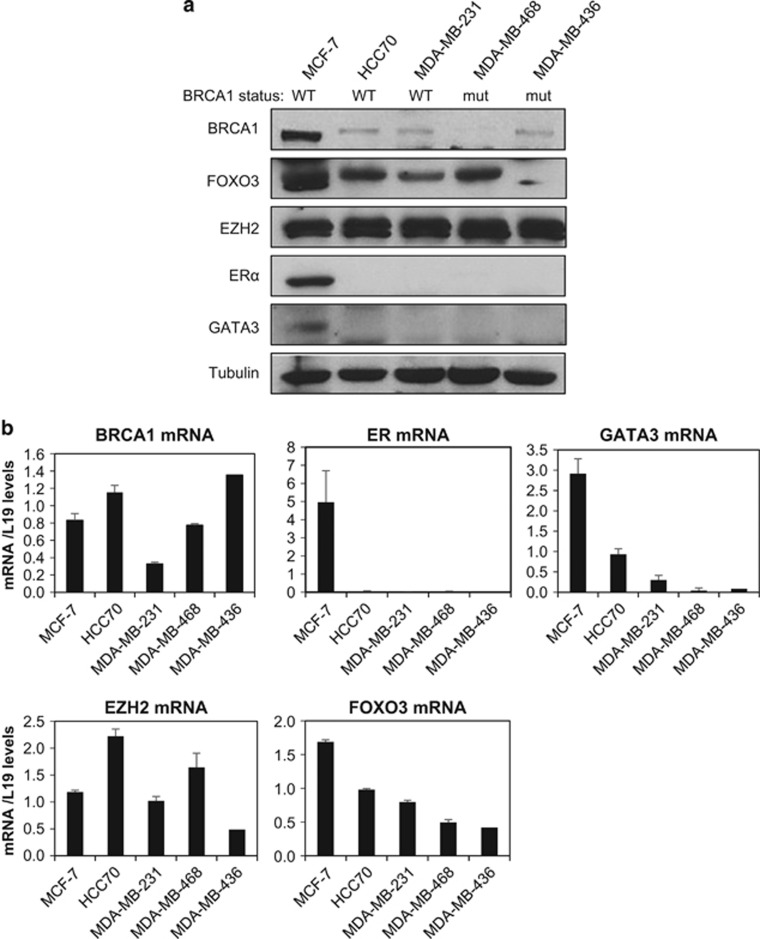
BRCA1 status correlates with FOXO3 expression in breast cancer cell lines. (**a**) Western blotting and (**b**) qRT–PCR analysis was performed on a panel of five different breast cancer cell lines including the luminal-type cell line MCF-7, which expresses wild-type BRCA1, basal-type cell lines HCC70, MDA-MB-231, MDA-MB-468 and MDA-MB-436, expressing either low or mutated BRCA1. (**a**) The expression of BRCA1, FOXO3, EZH2, ERα, GATA3 and Tubulin was examined by western blotting. (**b**) The experiments were repeated three times independently and qRT–PCR results were normalized against L19 mRNA levels and the results presented as bars representing mean±s.d.

To investigate the relationship between BRCA1 and FOXO3 expression, western blot analysis was performed on a panel of five breast cancer cell lines, which include the luminal-type MCF-7 cells with wild-type-competent BRCA1 and the basal-type lines HCC70, MDA-MB-231, MDA-MB-436 and MDA-MB-468 expressing either low or mutated BRCA1. Luminal markers, ERα and GATA3 were only expressed in MCF-7 but not in the basal-type breast cancer lines. Despite the great heterogeneity among the cell lines, there was strong correlation between the expression of wild-type BRCA1 and FOXO3. There was significant higher FOXO3 expression at both the mRNA and protein levels in MCF-7 compared with the basal-type cell lines (Figures 1a and b). Consistent with our previous results,^23^ the Polycomb protein EZH2 was found to be ubiquitously expressed in all five cell lines.

### BRCA1 regulates FOXO3 expression

**Figure 2:**
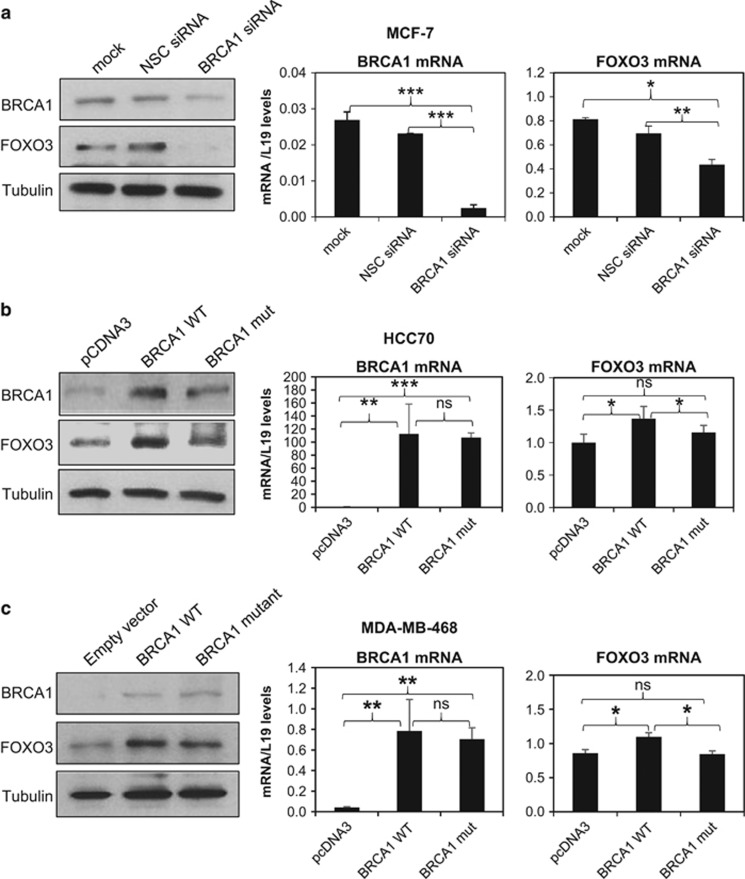
BRCA1 regulates FOXO3 expression in breast cancer cells. (**a**) BRCA1 depletion results in a significant reduction in FOXO3 expression levels in MCF-7 cells. Western blotting and qRT–PCR analysis was performed on MCF-7 cells transfected with BRCA1-specific siRNA pool or non-silencing control (NSC) siRNA pool for 48 h. FOXO3 expression was observed to be downregulated both at the protein and mRNA levels. For qRT–PCR analysis, the experiments were repeated three times independently and results normalized against L19 mRNA levels and expressed as mean±s.d. *P⩽0.05, **P⩽0.01 and ***P⩽0.001 by Students' *t*-test. (**b** and **c**) BRCA1 overexpression induces FOXO3 expression in the BRCA1-deficient HCC70 and MDA-MB-468 cell lines. (**b**) HCC70 and (**c**) MDA-MB-468 cells were transfected with the empty expression vector pcDNA3 or pcDNA3-HA-BRCA1 wild-type or mutant expression vector. Total protein was extracted from whole-cell lysates were extracted from these cells and analysed by western blotting with the indicated antibodies. FOXO3 and BRCA1 mRNA levels were also analysed by qRT–PCR, with results normalized with L19 mRNA levels. All qRT–PCR results presented as bars representing mean±s.d of three independent experiments in triplicates. *P⩽0.05, **P⩽0.01and ***P⩽0.001; NS not significant by Students' *t*-test (two-tailed).

The correlation between BRCA1 and FOXO3 expression in the panel of breast cancer cell lines led us hypothesize that BRCA1 regulates FOXO3 expression. To test this conjecture, BRCA1 was silenced in MCF-7 cells using a small interfering RNA (siRNA) pool. BRCA1 depletion resulted in a significant reduction in FOXO3 expression at both the protein and mRNA levels in MCF-7 cells (Figure 2a) compared with the non-silencing control and the mock transfected MCF-7 cells, supporting the notion that BRCA1 regulates FOXO3 expression. Consistently, overexpression of wild-type, but not a C61G mutant, BRCA1 in basal-type cell lines HCC70 (low BRCA1) and MDA-MB-468 (mutated BRCA1) significantly enhanced the expression of FOXO3 at both the protein and mRNA levels (Figures 2b and c, respectively). Taken together, these results suggest that BRCA1 regulates FOXO3 expression at least in part at the transcriptional level.

### 5′-Aza-dC treatment induces FOXO3 expression in basal-type cell lines

**Figure 3:**
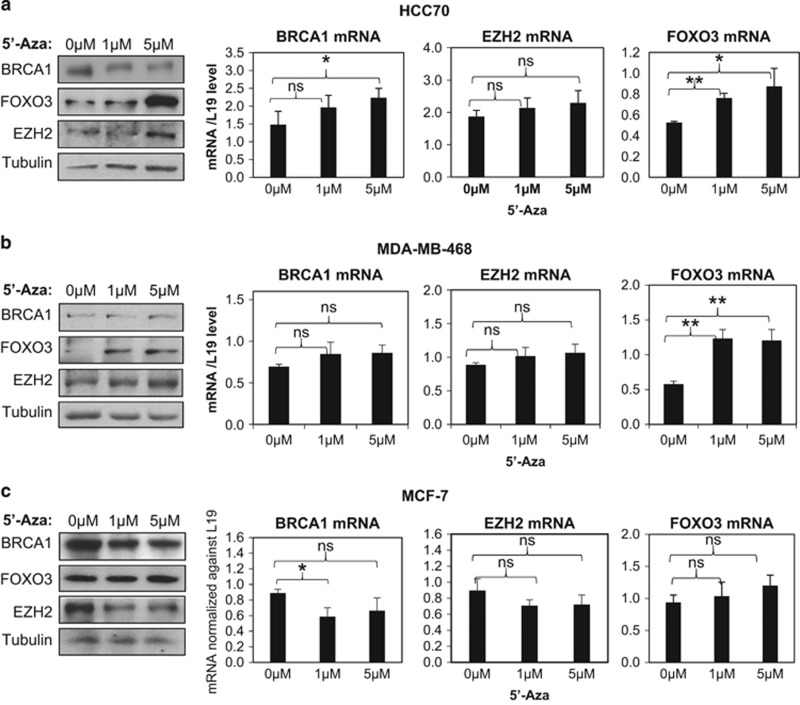
5′-Aza-dC treatment induces FOXO3 expression in basal-type cell lines. The basal type cell lines (**a**) HCC70 and (**b**) MDA-MB-468, as well as the luminal (**c**) MCF-7 cells were treated with 0, 1 and 5 μM of 5′-aza-dC for 72 h with culture medium changed every day. Total protein was extracted from these cells and analysed by western blotting with the indicated antibodies. In parallel, total RNA was also extracted and expression of BRCA1, EZH2 and FOXO3 mRNA was analysed by qRT–PCR. The experiments were repeated three times independently and qRT–PCR results were normalized against L19 mRNA levels and the results expressed as mean±s.d. *P⩽0.05 and **P⩽0.001; NS, not significant by Students' *t*-test.

To investigate further the relevance of *FOXO3* methylation in repressing FOXO3 expression in basal breast cancer, the basal-type cell lines HCC70 and MDA-MB-468, and the luminal-type cell line MCF-7 were treated with different amounts of 5′-aza-dC (0, 1 and 5 μM) for 72 h, and BRCA1, FOXO3 and EZH2 expression levels examined by both western blotting and quantitative reverse-transcriptase PCR (qRT–PCR). In HCC70, FOXO3 mRNA and protein expression was significantly induced after treatment with 1 and 5 μM of 5′-aza-dC for 72 h (both *P*<0.05, Students' *t*-test) (Figure 3a). In agreement, the FOXO3 expression was also increased after 5′-aza-dC treatment in MDA-MB-468 cells (Figure 3b). On the contrary, treatment of the luminal MCF-7 cells with 5′-aza-dC did not alter the expression levels of FOXO3 (Figure 3c), indicating that FOXO3 expression is repressed by DNA methylation in the absence of BRCA1. Collectively, these data provide evidence that *FOXO3* is methylated in basal subtype breast cancers, where BRCA1 is either mutated or depleted, highlighting the importance of BRCA1 in promoting FOXO3 expression through suppressing *FOXO3* methylation in luminal breast cancers.

### Inhibition or depletion of EZH2 induces FOXO3 expression

**Figure 4:**
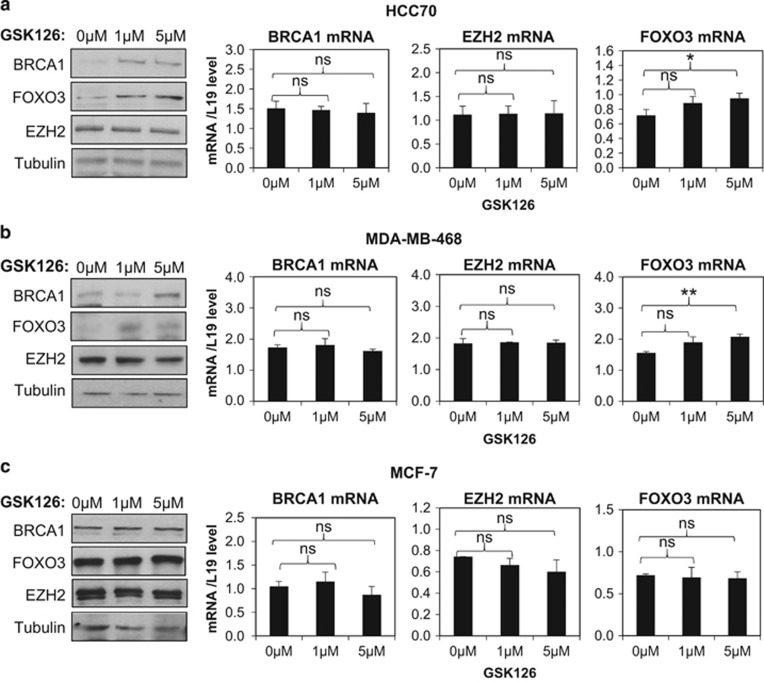
Inhibition of EZH2 induced FOXO3 expression in basal-type cell lines. The basal type cell lines (**a**) HCC70 and (**b**) MDA-MB-468, as well as the luminal (**c**) MCF-7 cells were treated with 0, 1 and 5 μM of the EZH2 inhibitor GSK126 for 72 h with culture medium changed every day. Total protein was extracted from these cells and analysed by western blotting with the indicated antibodies. In parallel, total RNA was also extracted and expression of BRCA1, EZH2 and FOXO3 mRNA was analysed by qRT–PCR. The experiments were repeated three times independently and qRT–PCR results were normalized against L19 mRNA levels and the results expressed as mean±s.d. *P⩽0.05 and **P⩽0.001; NS, not significant Students' *t*-test.

**Figure 5:**
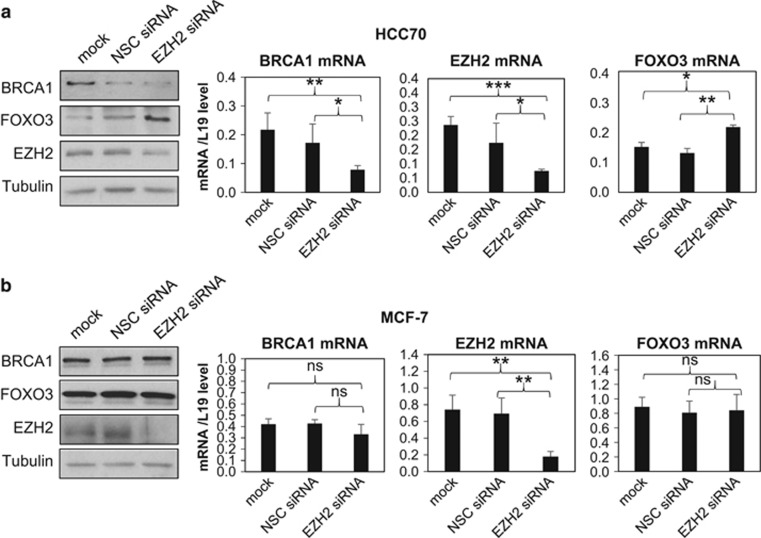
Depletion of EZH2 induced FOXO3 expression in HCC70 but not in MCF-7 cells. Western blotting and qRT–PCR analysis was performed on (**a**) HCC70 and (**b**) MCF-7 cells mock transfected or transfected with EZH2-specific siRNA pool or non-silencing control (NSC) siRNA pool for 48 h. Depletion of EZH2 by siRNA in HCC70 significantly induced FOXO3 expression but did not affect FOXO3 expression in MCF-7 cells. These experiments have been repeated three times and the representative western blottings were shown. qRT–PCR data were expressed as mean±s.d; *P<0.05, **P<0.01 and ***P<0.001; NS, not significant.

We next explored the mechanism by which BRCA1 negatively regulates *FOXO3* methylation and the cofactors involved. A previous study showed that BRCA1 interacts with EZH2 to inhibit its repression and the H3K27me3 on genes targeted by the PRC2 repressive complex in mouse embryonic stem and human breast cancer cells.^22^ We also showed recently that BRCA1 inhibits promoter methylation and chromatin silencing of *FOXA1* through binding to EZH2.^23^ These findings raised the possibility that BRCA1 promotes *FOXO3* expression through inhibiting EZH2 activity and histone lysine 27 trimethylation. To test this idea, we treated HCC70, MDA-MB-468 and MCF7 with GSK126, a highly selective *S*-adenosyl-methionine-competitive inhibitor of EZH2 methyltransferase activity,^24^ and studied its effect on FOX3a expression. The results showed that FOXO3 transcript and protein levels were significantly induced by treatment with 5 μM of GSK126 for 72 h in both the BRCA1-low basal-type cell HCC70 and BRCA1-mutated basal-type cell MDA-MB-468 (*P*<0.05, Students' *t*-test; Figure 4a and b, respectively). In comparison, FOXO3 expression was not affected by GSK126 in MCF7, which expresses high levels of wild-type BRCA1 (Figure 4c). This result suggests that EZH2 is involved in the repression of *FOXO3* expression in the BRCA1-deficient or -mutated cells but not in the BRCA1-competent cells. To further confirm this finding, EZH2 was depleted in the HCC70 and MCF7 cells. The knockdown efficiency of EZH2 after 72 h was confirmed by western blotting and qRT–PCR analysis. It was found that in HCC70 cells, EZH2 depletion significantly induced FOXO3 expression both at the protein and mRNA levels when compared with the non-silencing controls and the mock-transfected cells (Figure 5a). However, in MCF-7 cells, EZH2 knockdown did not affect FOXO3 expression (Figure 5b). Together, these data support the idea that EZH2 negatively regulates *FOXO3* transcription in BRCA1-deficient breast cancer cells, whereas this EZH2 activity is repressed by BRCA1 in BRCA1-competent cells.

### FOXO3 promoter is enriched with EZH2 and H3K27me3 in basal-type cells

**Figure 6:**
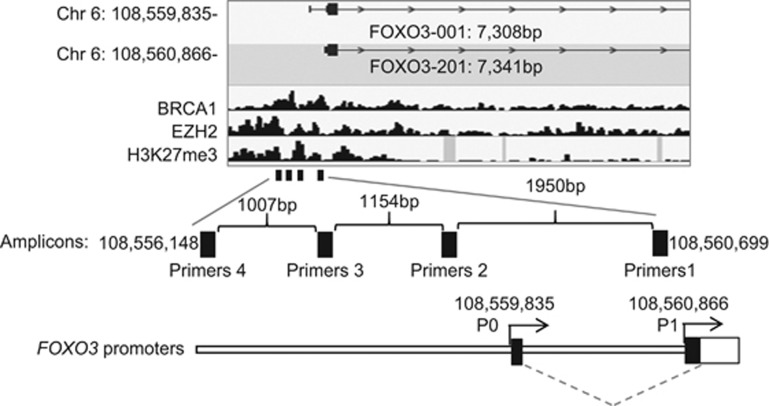
Schematic representation of the alignment of the binding profiles for EZH2, BRCA1 and H3K27me3, and the locations of the ChIP primers with the human *FOXO3* promoter. A schematic illustration of the human *FOXO3* promoter region, showing the two transcription start sites (Chr 6: 108,559,835- and Chr 6: 108,560,866-) (Top). ENCODE (the Encyclopedia of DNA Elements) project ChIP sequencing data of EZH2, BRCA1 and H3K27me3 binding in the liver carcinoma HepG2 (ATCC Number HB-8065) cells were used for predicting global genome-binding profiles for EZH2, BRCA1 and H3K27me3. The predicted binding profiles of EZH2, BRCA1 and H3K27me3 on the human *FOXO3* promoter are shown (below the FOXO3 promoter). The positions of the black boxes represent the amplicons of the designed ChIP primer pairs (primers 1–4; further below). The predicted binding profiles of EZH2, BRCA1 and H3K27me3, and the locations of amplicons from the designed ChIP primer pairs (primers 1–4) are aligned to the *FOXO3* promoter.

**Figure 7:**
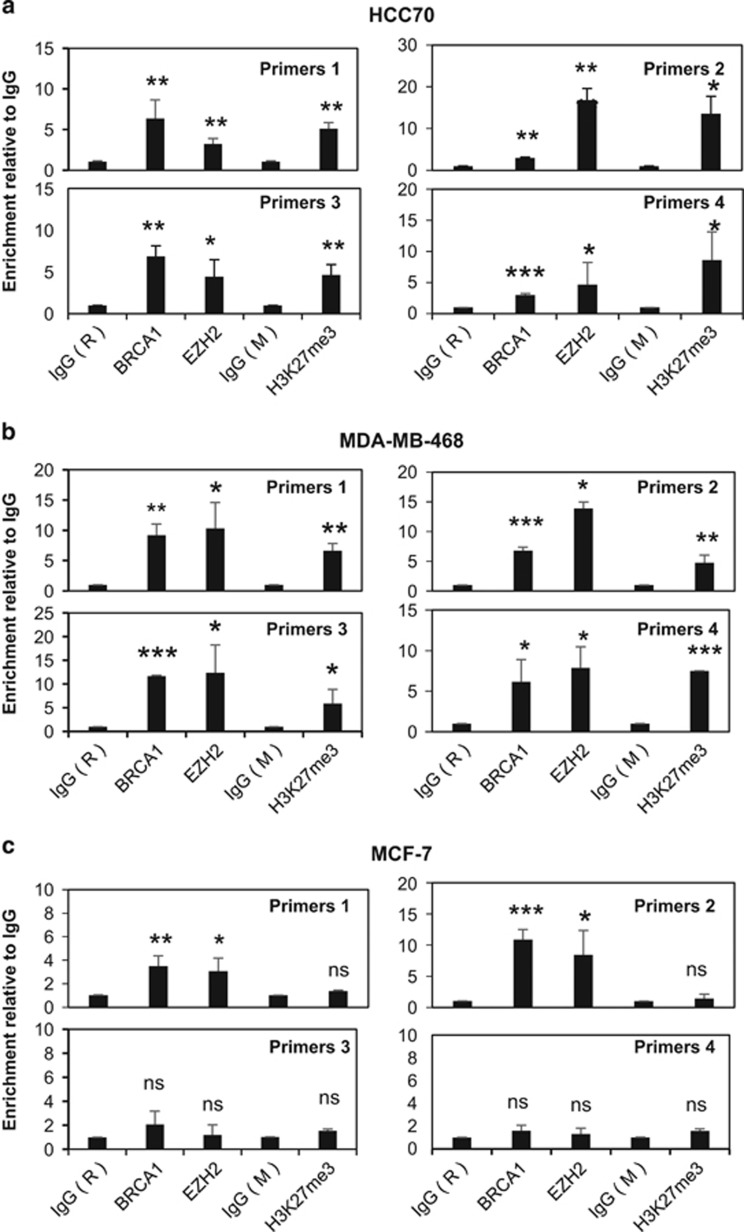
H3K27me3 is only enriched on the *FOXO3* promoter in the BRCA1-deficient HCC70 and MDA-MB-468 cells but not in the BRCA1-competent MCF-7 cells, qRT–PCR analysis of immunoprecipitated chromatin for the recruitment of BRCA1, EZH2 and H3K27me3 to the endogenous *FOXO3* promoter in HCC70, MDA-MB-468 and MCF-7 cells. (**a**) In HCC70, the ChIP–qPCR results showed that BRCA1, EZH2 and H3K27me3 were all recruited to the *FOXO3* promoter albeit BRCA1 at low levels. (**b**) BRCA1 (C61G mutant), EZH2 and H3K27me3 were recruited to the *FOXO3* promoter in MDA-MB-468 cells as revealed ChIP–qPCR analysis. (**c**) In MCF-7, BRCA1 and EZH2 were associated with the *FOXO3* promoter but H3K27me3 was not. The results were normalized to the amount of Input and compared with the IgG-negative controls. IgG (R), rabbit IgG-negative control; IgG (M), mouse IgG-negative control. These experiments were repeated three times independently and the qRT–PCR results presented as mean±s.d. *P<0.05, **P<0.01 and ***P<0.001; NS, not significant by Student's *t*-test (two-tailed).

**Figure 8:**
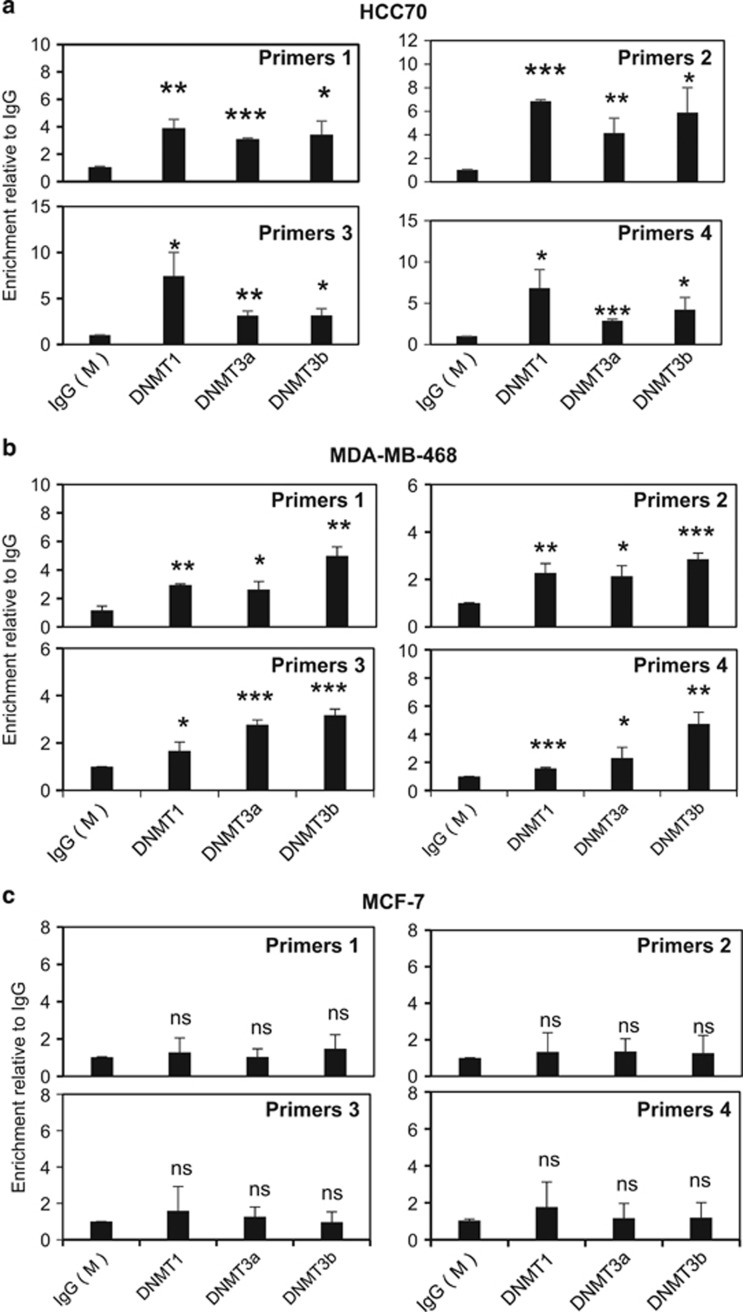
The methyltransferases DNMT1/3a/3b are only recruited to the *FOXO3* promoter in the basal-type HCC70 and MDA-MB-468 cells but not in MCF-7 cells. qRT–PCR analysis of immunoprecipitated chromatin for the recruitment of DNMT1, DNMT3a and DNMT3b to the endogenous *FOXO3* promoter in HCC70, MDA-MB-468 and MCF-7 cells. (**a**) In HCC70, the ChIP–qPCR results showed that DNMT1, DNMT3a and DNMT3b were all recruited to the *FOXO3* promoter. (**b**) DNMT1, DNMT3a and DNMT3b were recruited to the *FOXO3* promoter in MDA-MB-468 cells as revealed ChIP–qPCR analysis. (**c**) In MCF-7, neither of the DNMT1, DNMT3a and DNMT3b methyltransferases were associated with the endogenous *FOXO3* promoter. The results were normalized to the amount of Input and compared with the IgG-negative controls. These experiments were repeated three times independently and the qRT–PCR results presented as mean±s.d. *P<0.05, **P<0.01 and ***P<0.001; NS, not significant by Student's *t*-test (two-tailed).

Hitherto, our data indicated that BRCA1 inhibits EZH2 activity to suppress the silencing of *FOXO3*. Previously, EZH2 has been shown to cause H3K27me3 and DNA methylation at target genes through recruiting DNMTs including DNMT1, DNMT3a and DNMT3b.^25^ Collectively, these and our present findings suggest the possibility that EZH2 induces H3K27 trimethylation, which facilitates the recruitment of DNMTs to promote DNA methylation and silencing at the *FOXO3* promoter in BRCA1-deficient cells. To test this conjecture, we next investigated by chromatin immunoprecipitation (ChIP) analysis the occupancy of the human *FOXO3* promoter region by BRCA1, EZH2, DNMT1, DNMT3a, DNMT3b and H3K27me3 in the BRCA1-low HCC70 cells and BRCA1-mutated MDA-MB-468 cells, as well as the BRCA1-competent MCF-7 cells. *In silico* analysis of the transcription factor ChIP sequencing data sets in HepG2 cells available in the ENCODE project repository^26^ identified putative binding regions for EZH2, BRCA1 and H3K27me3, respectively, located on the *FOXO3* promoter (Figure 6). ChIP analysis was performed using primers designed to amplify across the EZH2-, BRCA1- and H3K27me3-binding regions upstream of the two transcription start sites on the *FOXO3* promoter (Figure 6). qRT–PCR results showed that the *FOXO3* promoter was enriched for BRCA1, EZH2 and H3K27me3 in the BRCA1-deficient HCC70 (Figure 7a) and MDA-MB-468 (Figure 7b) cells. In contrast, although both BRCA1 and EZH2 bound to the *FOXO3* promoter, there was no significant enrichment of H3K27me3 in the BRCA1-competent MCF-7 cells (Figure 7c). These results further support the notion that BRCA1 inhibits EZH2 to repress H3K27me3 on the *FOXO3* promoter. Consistently, ChIP analysis also revealed that DNMT1, DNMT3a and DNMT3b were enriched on *FOXO3* promoter in HCC70 (Figure 8a) and MDA-MB-468 (Figure 8b) but not in MCF-7 cells (Figure 8c). This indicates that DNA binding does not solely determine BRCA1 activity, which are regulated by multiple posttranslational mechanisms and its mutation status. In agreement, although both HCC70 and MCF-7 express wild-type BRCA1 and MDA-MB-468 has mutated BRCA1, MCF-7 is a luminal, while HCC70 and MDA-MB-468 are triple-negative breast cancer cell lines, which are more similar phenotypically to BRCA1-negative cells.

### BRCA1 represses the deposit of H3K27me3 and DNMTs to FOXO3 promoter

**Figure 9:**
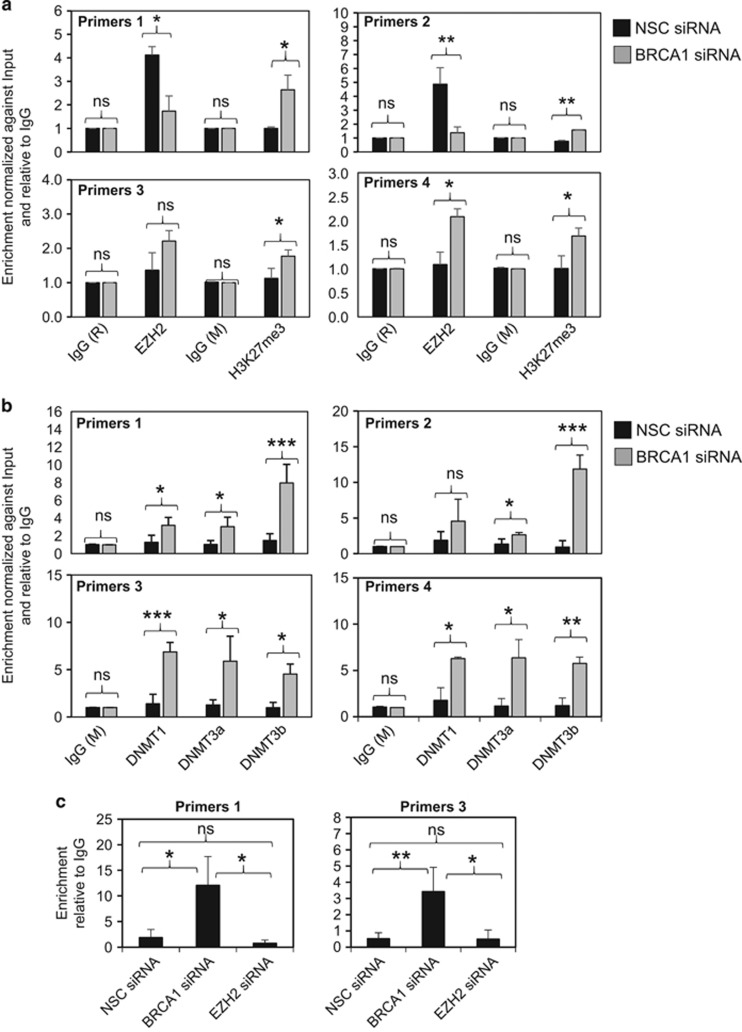
BRCA1 depletion causes the accumulation of H3K27me3, DNMT1/3a/3b and DNA methylation on the *FOXO3* promoter in MCF-7 cells. BRCA1 was transiently knocked down using specific siRNA pool in MCF-7 for 48 h. (**a**) MCF-7 cells transfected with BRCA1 and non-silencing control (NSC) siRNA pools independently were analysed for the accumulation of H3K27me3 on the endogenous *FOXO3* promoter by ChIP–qRT–PCR analysis. The results showed that despite the variable changes in EZH2 recruitment, there was always an increase in the accumulation of H3K27me3 marks on BRCA1 depletion. (**b**) MCF-7 cells transfected with BRCA1 and NSC siRNA pools independently were analysed for the recruitment of DNMT1/3a/3b to the endogenous *FOXO3* promoter by ChIP–qRT–PCR analysis. The results revealed that BRCA1 knockdown culminated in an increase in DNMT1/3a/3b recruitment. (**c**) MCF-7 cells transfected with BRCA1 and NSC siRNA pools independently were analysed for *FOXO3* promoter methylation by methylated DNA immunoprecipitation (MeDIP) qRT–PCR analysis. Despite the primers 2 and 4 consistently failed to generate reliable results, the results from primer sets 1 and 3 showed that BRCA1, but not EZH2, knockdown significantly enhanced *FOXO3* promoter methylation. The results were normalized to the amount of Input and compared with the IgG-negative controls. These experiments were repeated three times independently and the qRT–PCR results presented as mean±s.d. *P<0.05, **P<0.01 and ***P<0.001; NS, not significant by Student's *t*-test (two-tailed).

To establish further the role of BRCA1 in regulating the EZH2-induced deposition of histone mark H3K27me3 on the *FOXO3* promoter, BRCA1 was silenced using siRNA in MCF-7 cells, which express wild-type BRCA1. ChIP–qPCR analysis revealed that when compared with the non-silencing control siRNA-transfected controls, depletion of BRCA1 induced an accumulation of H3K27me3 marks on the *FOXO3* promoter, as detected by all four pairs of BRCA1/EZH2 primers (Figure 9a). Similarly, BRCA1 silencing also promoted the recruitment of DNMT1, DNMT3a and DNMT3b onto the *FOXO3* promoter, as detected by all four primer pairs (Figure 9b). These results are in line with our hypothesis that BRCA1 negatively regulates EZH2 to induce H3K27 trimethylation and the recruitment of DNMT1/3a/3b on the *FOXO3* promoter and gene silencing.

### BRCA1 represses FOXO3 promoter methylation in breast cancer

Our data demonstrated that BRCA1 suppresses the ability of EZH2 to recruit DNMT1/3a/3b to the *FOXO3* promoter, to repress FOXO3 expression. We next asked whether BRCA1 also represses methylation of the *FOXO3* promoter and used methylated DNA immunoprecipitation to study the methylation status of the *FOXO3* promoter in the absence or presence of BRCA1 or EZH2 depletion in the BRCA1 wild-type MCF-7 cells (Figure 9c). Our qRT–PCR showed that the levels of CpG methylation of FOXO3 genes increased on depletion of BRCA1 using siRNA, suggesting that BRCA1 represses the *FOXO3* promoter methylation and silencing. Notably, EZH2 depletion did not have a significant effect on *FOXO3* promoter methylation, probably due to the fact that the existing EZH2 is repressed by BRCA1 (Figure 9c).

### BRCA1 suppresses cell proliferation at least partially through FOXO3

To establish the functional significance of BRCA1–FOXO3 regulatory axis in breast cancer, we evaluated whether the anti-proliferative function of BRCA1 is mediated through FOXO3. To this end, HCC70 cells were transfected with either FOXO3 siRNA or non-silencing control siRNA together with the BRCA1 expression vector or the control empty vector (pcDNA3) (Supplementary Figure S1). Consistent with their tumour suppressor functions, FOXO3 depletion by siRNA accelerated cell proliferation when compared with the control cells transfected with non-silencing control siRNA and empty vector, whereas BRCA1 overexpression suppressed the cell proliferation (Supplementary Figure S1). Importantly, BRCA1 overexpression failed to effectively suppress cell proliferation when FOXO3 was depleted by siRNA, in particular at day 1. These results suggest that anti-proliferative function of BRCA1 was achieved, at least partially, through FOXO3.

### FOXO3 is hypermethylated and downregulated in BRCA1-mutated breast tumours

**Figure 10:**
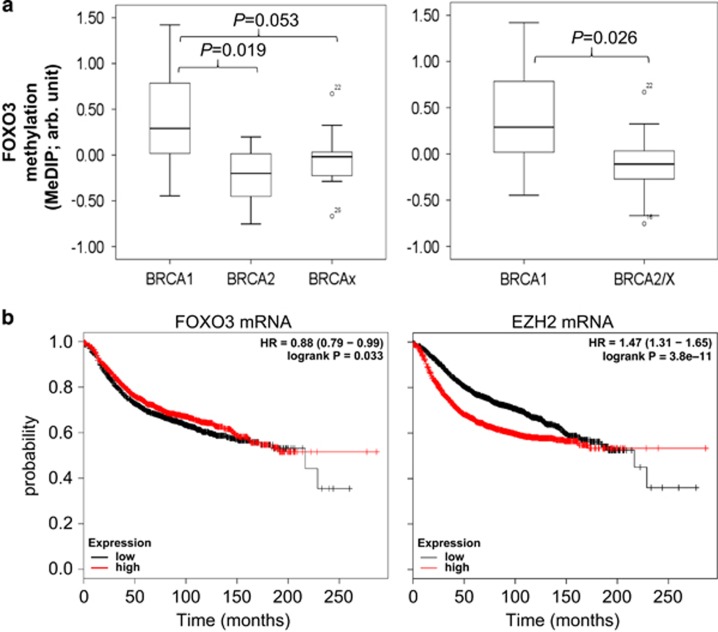
(**a**) *FOXO3* gene promoter is hypermethylated in BRCA1 mutation tumours. Frequency of *FOXO3* promoter methylation in clinical samples with mutations in BRCA1, BRCA2 and BRCAx tumours was analysed using the kConFab database. In 33 familial breast tumour samples, significant higher percentage of *FOXO3* promoter methylation was found in BRCA1-mutated tumours compared with BRCA2- or BRCAx-mutated tumour. Boxplots represent median (centre line), interquartile range (box) and 95th percentiles (whisker), and samples out with this range are represented as points. FOXO3 methylation scores were significantly higher in BRCA1-mutated samples when compared with BRCA2 or BRCAx (*P*=0.019 and *P*=0.053, respectively, Students' *t*-test) or BRCA2/x (*P*=0.026, Students' *t*-test). (**b**) Prognostic significance of FOXO3 and EZH2 mRNA in breast cancer. Examination of FOXO3 and EZH2 transcript expression in a previously published cohort (3455 breast cancer patients)^28^ revealed that both low FOXO3 and high EZH2 mRNA expression levels are very significantly associated with poor survival (*P*=0.033 and *P*=3.8 × 10^−1^, respectively, for overall survival, Kaplan–Meier analysis). The significance of both FOXO3 and EZH2in survival analyses provides further evidence for the involvement of both genes in breast cancer progression and drug response.

Having established that BRCA1 positively regulates FOXO3 expression through inhibiting the ability of EZH2 to mediate *FOXO3* methylation and silencing in human breast cancer cells, we next examined the association between FOXO3 methylation and BRCA1 mutation status in a methylated DNA immunoprecipitation data set from familial breast tumour samples collected by kConFab (The Kathleen Cuningham Foundation Consortium for Research into Familial Breast Cancer, Melbourne, Australia).^27^ There are 33 samples in the cohort, 11 of which harbour the BRCA1 mutation. Statistical analysis of the methylation profiles of *FOXO3* promoter in these familial breast cancers revealed that *FOXO3* methylation levels were significantly higher in BRCA1-mutated tumours, compared with BRCA2-, BRCAx- and BRCA2/x-mutated tumours (*P*=0.019, *P*=0.053 and *P*=0.026, respectively) (Figure 10a). In concordance, analysis of FOXO3 and EZH2 transcript levels in a previously published breast cancer cohort (3554 breast cancer patients)^28^ revealed that low FOXO3 and high EZH2 mRNA expression levels are very significantly associated with poor survival (*P*<0.033 and *P*=3.8 × 10^−11^, respectively, for overall survival, Kaplan–Meier analysis (Figure 10b). The association of both FOXO3 and EZH2 mRNA levels in survival analyses provides further evidence for the involvement of both genes in breast cancer progression.

**Figure 11:**
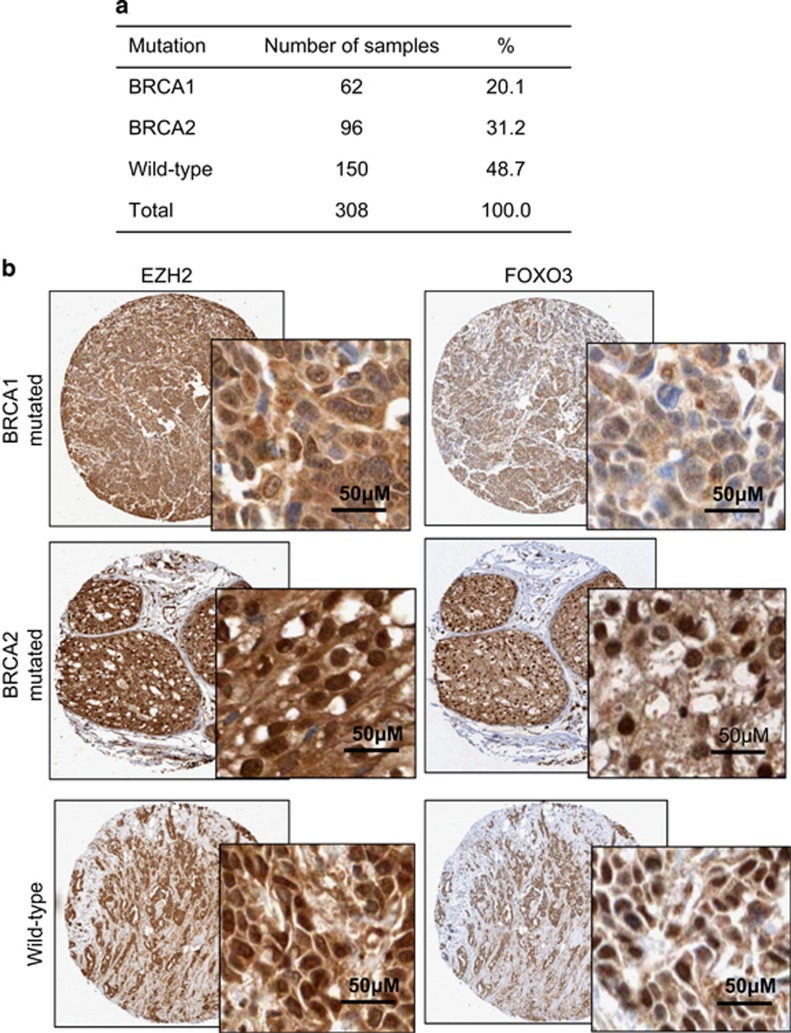
Immunostaining pattern of FOXO3 expression in breast cancers with different BRCA mutation status. (**a**) BRCA mutation makeup of tissue microarray constructed from 308 cases of Korean breast cancer samples. (**b**) Representative staining images of FOXO3 and EZH2 immunohistochemical staining of BRCA1-mutated, BRCA2-mutated or wild-type breast cancer samples. Images (original magnification, × 20); insets (original magnification, × 100).

**Figure 12:**
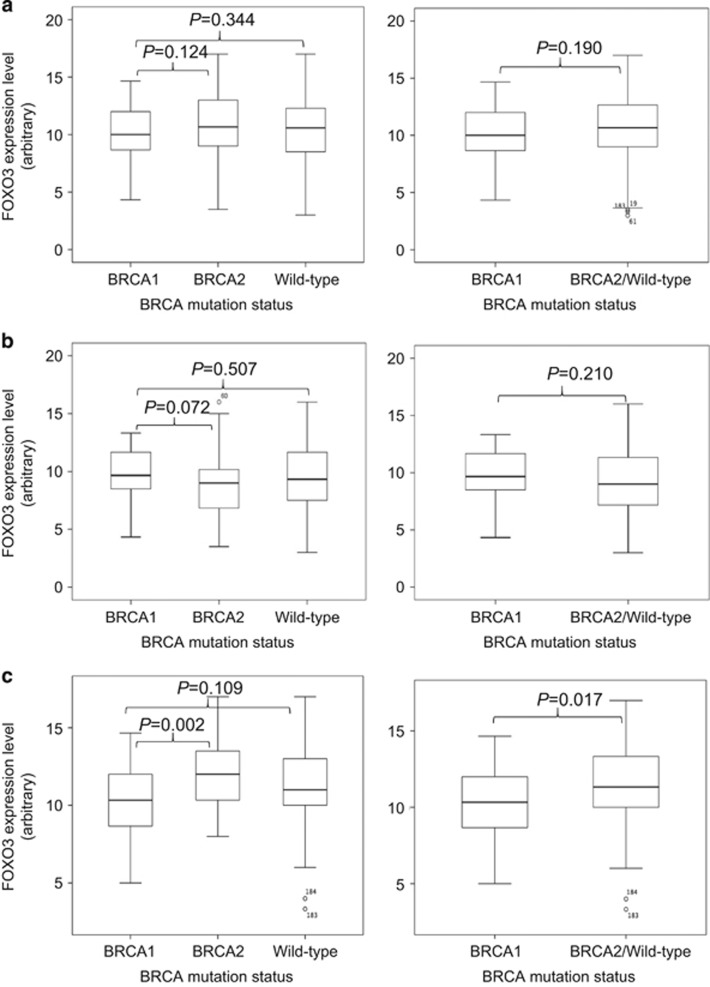
FOXO3 and EZH2 expression levels in breast cancers with different BRCA mutation. (**a**) Comparison of FOXO3 expression levels with different BRCA mutation status by Mann–Whitney *U*-rank test using all samples. (**b**) Comparison of FOXO3 expression levels with different BRCA mutation status using samples that express low levels of nuclear EZH2 by Mann–Whitney *U*-rank test. (**c**) Comparison of FOXO3 expression levels with different BRCA mutation status compared using samples that express high levels of nuclear EZH2 by Mann–Whitney *U*-rank test.

To further affirm our results, we also investigated the correlation between BRCA1 mutation status and FOXO3 protein expression levels in human samples by immunohistochemical staining on tissue microarray constructed from 308 Korean breast cancer cases with known *BRCA1* status (Figure 11a). The representative images of the immunohistochemical staining of BRCA1-mutated, BRCA2-mutated and wild-type samples are shown (Figure 11b). It was found that, although not statistically significant, the mean FOXO3 protein expression levels were lower in BRCA1-mutated samples in comparison with BRCA2-mutated, non-mutated or non-BRCA1-mutated (that is, both BRCA2-mutated and wild-type samples) (Figure 12a, Mann–Whiney *U*-rank test, *P*=0.124, *P*=0.344 and *P*=0.19, respectively). As EZH2 could also affect FOXO3 expression, especially in BRCA1-mutated cells, we therefore investigated the correlations between FOXO3 expression and BRCA mutation status in samples adjusted for their EZH2 expression levels. As shown in Figure 11b, EZH2 was expressed in both the cytoplasm and the nucleus. The expression levels of EZH2 in the cytoplasm were similar among all the samples with almost all the cell cytoplasm showing intermediate intensity, whereas the nuclear EZH2 expression varied between samples. Moreover, EZH2 is mainly functionally active in the nucleus to catalyse methylation of H3K27 and, therefore, only nuclear EZH2 expression was taken into account when analysing the data. In patients who expressed low nuclear EZH2 (using median score as cutoff), there was no significant difference in terms of FOXO3 expression levels between BRCA1 and BRCA2-mutated samples, between BRCA1 and wild-type samples, and between BRCA1 and non-BRCA1-mutated samples (Figure 12b, Mann–Whitney *U*-rank test, *P*=0.072, *P*=0.507 and *P*=0.21, respectively). In comparison, in patients who expressed high nuclear EZH2, FOXO3 expression was significantly lower in BRCA1-mutated samples when comparing with BRCA2-mutated samples, and with non-BRCA1-mutated samples (Figure 12c, Mann–Whitney *U*-rank test, *P*=0.002 and *P*=0.017, respectively). These results indicate the regulation of FOXO3 by BRCA1 via EZH2. Collectively, these *in vivo* data provided strong evidence for the *in vitro* finding that BRCA1 positively regulates FOXO3 expression by suppressing the function of EZH2, to promote *FOXO3* methylation and silencing.

## Discussion

The breast cancer susceptibility protein BRCA1 is an important tumour suppressor in breast cancer. Mutations in BRCA1 predispose women to a higher lifetime risk of breast and ovarian cancer.^29^ BRCA1 is implicated in mammary epithelial cell differentiation and its deficiency is often associated with basal-like breast cancer subtype.^30^ Although it has been reported that BRCA1 imposes its tumour suppressive role by regulating DNA damage repair and cell cycle checkpoint, the exact reason whereby BRCA1 deficiency or mutation causes more aggressive breast cancer phenotype remains largely unknown.

In this study, we show that BRCA1 prevents and reverses *FOXO3* suppression through suppressing the PRC2 methyltransferase EZH2. FOXO3 expression was restored in basal but not in BRCA1-competent MCF-7 cells following treatment with 5′-aza-dC, supporting our hypothesis that FOXO3 is silenced by hypermethylation in the basal BRCA1-deficient cell lines. Our data also reveal that *FOXO3* gene expression is negatively regulated by EZH2 in basal cell lines, where *BRCA1* is downregulated or mutated, but not in the BRCA1-competent MCF-7 cells, affirming that BRCA1 represses the ability of EZH2 to suppress FOXO3 expression. Moreover, chemical inhibition or siRNA-mediated depletion of EZH2 enhanced FOXO3 expression in basal cell lines and not in MCF-7 cells, suggesting that in the presence of BRCA1, EZH2 is functionally inactive and is unable to suppress FOXO3 expression. Consistently, there has been evidence suggesting the regulation of *FOXO3* expression by DNA methylation. For example, FOXO3 expression is regulated by its DNA methylation status in mouse embryonic fibroblasts^31^ and hypomethylating agents including Azacytidine and Decitabine can restore FOXO3 in acute myeloid leukaemia patients.^32^ *FOXO3* has also been identified as one of the target genes regulated by EZH2-H3K27me3-dependent transcriptional network in hepatocellular carcinoma.^33^ On the contrary, FOXO3 has also been reported to be a transcriptional regulator of DNMT3b in lung cancer.^34^

EZH2, a key subunit in the PRC2, has been shown to epigenetically suppress target gene expression through modulating both histone and DNA methylation. Accordingly, in addition to mediating the trimethylation of H3K27, an epigenetic mark for transcriptional silencing, EZH2 also directly recruits DNMTs, to induce DNA methylation and gene silencing. As BRCA1 has been shown to be able to bind EZH2 and negatively regulate PRC2 complex activity,^22^ and as we have previously shown that both BRCA1 and DMNT3 bind to EZH2 but do not exist in the same complexes in breast cancer cells,^23^ these lead us to propose that BRCA1 can promote the transcription of *FOXO3* indirectly through binding to EZH2 subunit of the PRC2 complex, thereby restraining its methyltransferase activity. In agreement, our ChIP assays showed that DNMT1/3a/3b and the transcriptional repressive histone mark H3K27me3 are recruited to the promoter region of *FOXO3* in BRCA1-low and -mutated breast cancer cell lines but not in BRCA1-competent MCF-7 cells. Furthermore, depletion of BRCA1 by siRNA in MCF-7 induces the deposition of H3K27me3 on the *FOXO3* promoter.

These *in vitro* findings are further corroborated by the *in vivo* correlation of BRCA1 mutation with FOXO3 promoter methylation and protein expression. FOXO3 methylation levels were significantly higher in BRCA1-mutated tumours compared with BRCA2, BRCAx (non-BRCA1/2) and non-BRCA1-mutated (BRCA2/x) tumours (Figure 12). More interestingly, in patients who expressed low EZH2, there are no significant differences in FOXO3 protein expression levels when comparing BRCA1-mutated samples with BRCA2-mutated, wild-type or non-BRCA1-mutated samples. However, there are statistically significant differences in FOXO3 expression levels between BRCA1-mutated and BRCA2-mutated or non-BRCA1-mutated samples (Figure 12). These results indicate that BRCA1 is not able to regulate FOXO3 expression when EZH2 expression or activity is depleted, suggesting EZH2 is a key intermediate in the control of FOXO3 by BRCA1. In other words, the role of BRCA1 in positively regulating FOXO3 expression is mediated through EZH2, consistent with the previous *in vitro* results showing that BRCA1 promotes FOXO3 expression through relieving the suppressive function of EZH2.

FOXO3 is a *bona fide* pleiotropic tumour suppressor that negatively regulates cell proliferation and cancer progression by regulating the expression of genes involved in differentiation, apoptosis, cell cycle regulation, oxidative stress response, DNA damage repair, metastasis and angiogenesis.^35^ FOXO3 can also antagonize functions of FOXM1, which is a potent oncogene that has a central role in promoting cell proliferation, migration, invasion, angiogenesis, stem cell renewal and DNA damage repair, processes which contribute to cancer initiation, progression and drug resistance.^12, 35^ As a result, FOXO3 expression is a good prognostic marker for breast cancer,^14, 36^ except when it is deregulated and resides constitutively in the nucleus.^37^ FOXO3 is also a molecular target of multiple clinically available or potential anti-cancer therapeutics and, therefore, its deregulation could culminate in drug resistance.^38^ Doxorubicin treatment could cause the phosphorylation of FOXO3 by stress-activated p38 mitogen-activated protein kinase and subsequently nuclear localization and activation of FOXO3.^13^ Another recent study showed that low dose of metformin, which is the active metabolite of a topoisomerase-1 inhibitor derivative, suppresses breast and ovarian cancer growth both *in vitro* and *in vivo* in a FOXO3-dependent manner.^39^ As FOXO3 is such an crucial tumour suppressor and therapeutic drug target, it is pertinent to appreciate how FOXO3 expression is regulated normally and also deregulated in cancer. Past studies have reported that the FOXO3 is primarily regulated by multiple kinases that could phosphorylate FOXO3, which subsequently lead to nuclear exclusion and ubiquitination/degradation in the cytoplasm.^35, 40^ This study reports a novel regulatory mechanism of FOXO3 by BRCA1 in breast cancer where BRCA1 indirectly regulates FOXO3 expression through interfering with EZH2-H3K27me3 deposition onto the *FOXO3* promoter and its DNA methylation. The finding that overexpression of FOXO3 mRNA levels is a good prognostic factor in breast cancer (Figure 10b) further supports the importance of FOXO3 regulation at the transcriptional level in breast cancer development.

This could be important in breast cancer progression and contribute to the understanding of tumour suppressive role of BRCA1. In agreement, our result also show that BRCA1 suppresses proliferation of basal type breast cancer cell line HCC70, at least partially through FOXO3, as depletion of FOXO3 by siRNA compromised the cell proliferation suppression induced by BRCA1 overexpression. The results suggest that the tumour suppressive role of BRCA1 is partially achieved by regulating FOXO3 expression in breast cancer. In line with our findings, recent studies have suggested that FOXO3 expression is regulated by its DNA methylation status in mouse embryonic fibroblasts^31^ and hypomethylating agents can reactivate FOXO3 in acute myeloid leukaemia.^32^ Consistently, another recent study has also identified *FOXO3* as one of the target genes controlled by the EZH2-H3K27me3-dependent transcriptional network in hepatocellular carcinoma.^33^ In concordance, a recent Phase I/Ib trail of olaparib and carboplatin in BRCA1 or BRCA2 mutation-associated breast or ovarian cancer has revealed that FOXO3 expression is associated with responsiveness.^41^ Thus, clinically approved inhibitors of DNMT1 and DNMT3a/b, such as 5-Azacytidine (azacitidine) and 5-azadeoxycytidine (decitabine)^42, 43^, or small molecule inhibitors of EZH2 activity, including GSK126 and DZNep (3-deazaneplanocin),^24, 44^ may be used to restore FOXO3, to enhance the efficacy of chemotherapeutic drugs in BRCA1/2 mutation-associated breast or ovarian cancer. In addition, the expression levels of FOXO3 and FOXA1 may also be useful biomarkers to molecularly classify BRCA1-mutated breast cancers.

To summarize, the current study demonstrates that BRCA1 positively regulates FOXO3 expression through inhibiting the activity of ESH2 in breast cancer, whereas depletion or mutation of BRCA1 would lead to restoration of the ability of EZH2 to recruit DNMT1/3a/3b methyltransferases and H3K27me3 histone marks, to mediate methylation and silencing of the *FOXO3* gene. These findings contribute to better understand the tumour suppressor role of BRCA1 and the regulation of another tumour suppressor FOXO3 in breast cancer, and suggest FOXO3 as a diagnostic marker and therapeutic target for BRCA1-deficient basal-like breast cancer. Thus, this study has potential important diagnostic and therapeutic implications for BRCA1 functional deficient breast cancer.

## Materials and methods

### Cell culture

The human breast carcinoma cell lines MCF-7, MDA-MB-231, MDA-MB-436, MDA-MB-468 and HCC70 originated from the American Type Culture Collection ((LGC standards, Middlesex, UK) and were authenticated by Cancer Research UK (London, UK). See also Supplementary Materials and Methods.

### FuGENE6 transfection

Cells were seeded into six-well plates or 150-mm dishes, to achieve ~60% confluency before transfection. Plasmid DNA was transfected using FuGENE 6 (Roche Diagnostics, West Sussex, UK) in a 3:1 ratio following manufacturer's instructions. The pcDNA3-HA-BRCA1 wild-type expression plasmids have previously been described^45^ and were obtained from Dr David M Livingston (Harvard Medical School, Boston, MA, USA). The BRCA1 mutant used is the cancer-predisposing mutation C61G disrupts homodimer formation in the NH2-terminal BRCA1 RING finger domain. It was generated by site-directed mutagenesis (by Quickchange Mutagenesis kit; #200521, Agilent Technologies LDA UK Limited, Gangnam-gu, Seoul, Korea) from the BRCA1 wild-type expression vector.

### Gene silencing with siRNAs

All siRNAs for the work were ON-TARGET*plus* SMARTpool siRNA purchased from Dharmacon Thermo Scientific (Lafayette, CO, USA). The SMARTpool siRNAs used in this study were: siBRCA1 (L-003461-00), siEZH2 (L-004218-00), siFOXO3 (L-003007-00-) and the ON-TARGET*plus* Non-Targeting Pool (D-001810-10). The Dharmacon SMARTpool siRNA consists of at least four different target-validated siRNA species designed to increase target specificity and to minimize off-target effects. All siRNA pools were resuspended to 20 μM in 1 × siRNA buffer before use. siRNAs or non-targeting controls were delivered into the cells seeded in six-well plates or 150-mm dishes by using Oligofectamine (Invitrogen, Paisley, UK) following the manufacturer's instruction. Cells were collected 72 h after transfection.

### Western blot analysis

Cells were collected for western blot analysis as described.^46^ Protein concentration was determined by BCA protein assay (Bio-Rad, Hemel Hempstead, UK). Twenty micrograms of protein were separated by SDS–polyacrylamide gel electrophoresis, transferred to nitrocellulose membrane and hybridized with the following antibodies at 4 °C for overnight: BRCA1 (1:1000, Millipore; 07-434, Watford, UK)^23^, EZH2 (1:1000, Diagenode, Seraing/Ougrée, Belgium; C15410039), ERα (1:1000, Santa-Cruz, Insight Biotechnology Ltd, Wembley, UK; sc-7207), β-tubulin (1:1000, Santa-Cruz); FOXO3 (1:3000, Millipore) and GATA3 (1:1000, Santa-Cruz; H-48). On the second day, the membranes were washed three times with TBST, incubated with horseradish peroxidase-conjugated secondary antibody (1:30000, DAKO, Ely, UK) for 1 h. The chemilluminance signals were detected by incubating the membranes with ECL substrate (Perkin Elmer, Seer Green, UK) and exposed to X-ray films (GE Healthcare, Amersham, UK).

### RNA extraction, reverse transcription and real-time quantitative PCR

For RNA extraction, reverse transcription and real-time quantitative PCR, see Supplementary Materials and Methods.

### Chromatin immunoprecipitation

Forty microlitres of Dynabeads Protein A/G was washed with 200 μl of TSE I buffer for three times and diluted with 40 μl of TSE I buffer. Four micrograms of antibodies against BRCA1 (Millipore, 07-434), EZH2 (Diagenode; C15410039), DNMT1 (Abcam; ab87656), DNMT3a (Abcam; ab2850), DNMT3b (Abcam; ab13604), H3K27me3 (Abcam; ab6002) and rabbit/mouse IgG negative control (DAKO) were used for each ChIP experiment. All ChIP data were originally acquired as % of input and further normalized with the values for IgG controls. For details, see Supplementary Materials and Methods Quantitative real-time PCR were performed the using primers listed in Supplementary Figure S2.

### Sulforhodamine B assay

For sulforhodamine B assay, see Supplementary Materials and Methods.

### Tissue microarray, immunohistochemistry and staining scoring

The tissue microarray for analysis of FOXO3 expression and BRCA mutation status was from the Asan Medical Center, University of Ulsan College of Medicine, Seoul, Korea. The tissue microarray contains 308 cases of breast cancer cases including 62 cases with BRCA1 mutation, 96 cases with BRCA2 mutation and 150 cases of wild-type BRCA with patient consent. For immunohistochemistry and staining scoring, see Supplementary Materials and Methods. To avoid subjectivity in evaluation, staining intensity and percentage was scored by two independent individuals in a semi-quantitative way and the average was taken. Cytoplasm and nucleus expression of FOXO3 and EZH2 was scored as previously described.^37^

### Statistical analysis

Students' *t*-test was used to evaluate the difference between treatment and control group or between non-transfected samples and transfected samples. The correlations between FOXO3 expression levels and BRCA mutation status were studied by Mann–Whitney *U*-rank test in SPSS (IBM, version 17, Portsmouth, UK). *P*-values <0.05 were considered statistically significant.
