# Supplementary material for: BRCA1 positively regulates FOXO3 expression by restricting FOXO3 gene methylation and epigenetic silencing through targeting EZH2 in breast cancer
Source: Oncogenesis. 2016 Apr 4;5(4):e214–. doi: 10.1038/oncsis.2016.23 (PMC4848836; doi:10.1038/oncsis.2016.23)
Supplement: Supplementary Information [file oncsis201623x1.doc]

**The COHBRA study group**

Beom Seok Kwak, Byeong-Woo Park, Byung Ho Son, Byung-In Moon, Cha Kyong Yom, Chan Heun Park, Chan Seok Yoon, Chang Hyun Lee, Dae Sung

Yoon, Dong-Young Noh, Doo Ho Choi, Eundeok Chang, Eun-Kyu Kim, Eunyoung Kang, Hae Kyung Lee, Hai-Lin Park, Hyde Lee, Hyeong-Gon Moon, Hyun-Ah Kim, Il-Kyun Lee, Jeong Eon Lee, Jihyoun Lee, Jong Won Lee, Jong-Han Yu, Joon Jeong, Jung Han Yoon, Jung-Hyun Yang, Keumhee Kwak, Ki-Tae Hwang, Ku Sang Kim, Lee Su Kim, Min Hee Hur, Min Ho Park, Min Hyuk Lee, Myung Chul Chang, Nam Sun Paik, Sang Ah Han, Sang Seol Jung, Sang Uk Woo, Se Jeong Oh, Sehwan Han, Sei Joong Kim, Sei-Hyun Ahn, Seok-Jin Nam, Seung Sang Ko, Sung Hoo Jung, Sung Soo Kang, Sung Yong Kim, Sung-Won Kim, Tae Hyun Kim, Tae Wan Won, Tae Woo Kang, Wonshik Han, Woo-Chul Noh, Yong Lai Park, Yongsik Jung, Young Jin Suh, Young Tae Bae, Young Up Cho, Young-Ik Hong, Sue K. Park, Yoon Joo Jung, Su Yun Choi, Young Bum Yoo, Soo-Jung Lee.
