# Supplementary material for: BRCA1 positively regulates FOXO3 expression by restricting FOXO3 gene methylation and epigenetic silencing through targeting EZH2 in breast cancer
Source: Oncogenesis. 2016 Apr 4;5(4):e214–. doi: 10.1038/oncsis.2016.23 (PMC4848836; doi:10.1038/oncsis.2016.23)
Supplement: Supplementary Information [file oncsis201623x2.doc]

**Supplementary Materials and Methods**

**Cell culture**

The human breast carcinoma cell lines MCF-7, MDA-MB-231, MDA-MB-436, MDA-MB-468 and HCC70 originated from the American Type Culture Collection ((LGC standards, Middlesex, UK) and were authenticated by Cancer Research UK (London, UK). MCF7, MDA-MB-231 and MDA-MB-468 cell lines were maintained in Dulbecco’s modified eagle’s medium (DMEM) (Sigma-Aldrich, Poole, UK) supplemented with 10% foetal calf serum (FCS) (First Link Ltd, Birmingham, UK), 4 mM glutamine and 100 U/ml penicillin/streptomycin (Sigma-Aldrich, UK). MDA-MB-436 cells were maintained in Leibovitz’s L-15 medium (Gibco, Life Technologies; 11415-049) supplemented with 10% horse serum (Invitrogen), while HCC70 cells were cultured in RPMI 1640 (Gibco:21765-029) supplemented with 10% foetal calf serum (FCS), 4 mM glutamine and 100 U/ml penicillin/streptomycin (Sigma-Aldrich, UK). All the cells were cultured in 37 ^0^C incubator supplied with 5% CO_2_.

**Chromatin immunoprecipitation (ChIP)**

40 μl of Dynabeads Protein A/G was washed with 200 μl of TSE I buffer for three times and diluted with 40μl of TSE I buffer. 4μg of antibodies against BRCA1 (Millipore, 07-434), EZH2 (Diagenode; C15410039), DNMT1 (Abcam; ab87656), DNMT3a (Abcam; ab2850), DNMT3b (Abcam; ab13604), H3K27me3 (Abcam; ab6002), rabbit/mouse IgG negative control (DAKO, Ely, UK) were first separately diluted in Buffer D, mixed with diluted Dynabeads and then rotated O/N at 4^0^C Cells cultured in 10 mm dish with 90% confluency were crosslinked with 1% formaldehyde for 10 min, rinsed with ice-cold PBS, incubated with 2.5 M glycine for 5 min and harvested with 2ml of scrapping buffer. After a sequential wash with PBS, Buffer I and Buffer II, cell pellet was resuspended in 300 μl of Lysis buffer and sonicated in cold room. Supernatant was then diluted in 300 μl of Buffer D from which 100 μl was taken as INPUT. 200μl of cell lysate was mixed with prepared Dynabeads and rotated O/N at 4°C. After a sequential wash with TSE I, TSE II and TE buffer, 100 μl of elution buffer was added to the Dynabeads and the mixture was rotated at RT for 1 h. Eluted sample was collected in eppendorf and the Dynabeads was re-eluted with another 100 μl of elution buffer. The samples were crosslinked by incubating at 65°C O/N. PCR Purification Kit (Qiagen) was then used to purify DNA following manufacture’s instruction. Quantitative real-time PCR were performed the using primers listed in Supplementary Figure S2.

**Immunohistochemistry and Staining Scoring**

The TMA sections were deparaffinized and rehydrated by incubation with xylene and decreasing concentrations of ethanol. Antigen retrieval was performed by heating using pressure cooker in EDTA buffer. The slides were immersed into 3% H_2_O_2_/methanol for 10 min at room temperature to quench endogenous peroxidase. After rinsing in 0.05% Tween in PBS (PBST) twice, anti-FOXO3 (1:850, Millipore) or anti-EZH2 (1:300, Diagenode) was added to each section and incubated at 4°C overnight. The slides were then washed in PBST and incubated with DAKO EnVision+System-HRP-labelled Polymer Anti-Rabbit at room temperature in dark for 30 min. After washing, Chromogen DAB/substrate reagent was added onto the slides and the slides incubated further for several minutes. Finally, the slides were dehydrated and mounted. Aperio ScanScope ® system (Aperio technology, USA) was used to visualize and assess for FOXO3 and EZH2 expression. The TMA slides were scanned by ScanScope scanners and individual stained TMA spots were assessed in computer screen with the use of Aperio’s image viewer, ImageScope.

To avoid subjectivity in evaluation, the intensity and percentage of the staining were scored by two independent individuals in a semi-quantitative way and the average was taken. In brief, the intensity and percentage of the staining were assessed separately in cytoplasm and nucleus. The intensity was scored as 0= none, 1= weak, 2=moderate, 3=strong. The percentage was scored as 1= less than 25%, 2= 25% to 50%, 3= 50% to 75% and 4= more than 75%. Cytoplasm/nucleus score was calculated as intensity * percentage and total score was calculated as cytoplasm score + nucleus score.

**Sulforhodamine B (SRB) assay**

Cells were seeded in 96-wells prior to the assay. On the day of harvesting, the cells were firstly fixed in 40% trichloroacetic acid at 4^0^C for 1 hour, washed three times with tab water and stained with SRB solution at room temperature for 1 hour. After staining, the cells were washed with 1% acetic acid and dried overnight. The protein-bound dye was dissolved in 10mM Tris base solution and the absorbance was measured at 492 nm using Tecan Microplate reader.

**RNA extraction, Reverse transcription and Real-time quantitative PCR (RT-qPCR)**

Total RNA was extracted using RNasey kit (QIAGEN) and up to 2µg of total RNA was reversely transcribed into cDNA using SuperScript Transcriptase III (Invitrogen) following manufacturer’s protocol. Amount of transcripts were quantified by absolute quantification (AQ) using Power SYBR Green PCR Master Mix (Applied Biosystems, Life Technologies, UK) and analysed by ABI7900 Sequence Detection System (Applied Biosystems). Housekeeping gene L19 was used as internal control for normalization. Sequences of primers used are listed in Supplementary Fig. S2.
