# Supplementary material for: BRCA1 positively regulates FOXO3 expression by restricting FOXO3 gene methylation and epigenetic silencing through targeting EZH2 in breast cancer
Source: Oncogenesis. 2016 Apr 4;5(4):e214–. doi: 10.1038/oncsis.2016.23 (PMC4848836; doi:10.1038/oncsis.2016.23)
Supplement: Supplementary Figure 1 [file oncsis201623x3.ppt]

## Slide 1
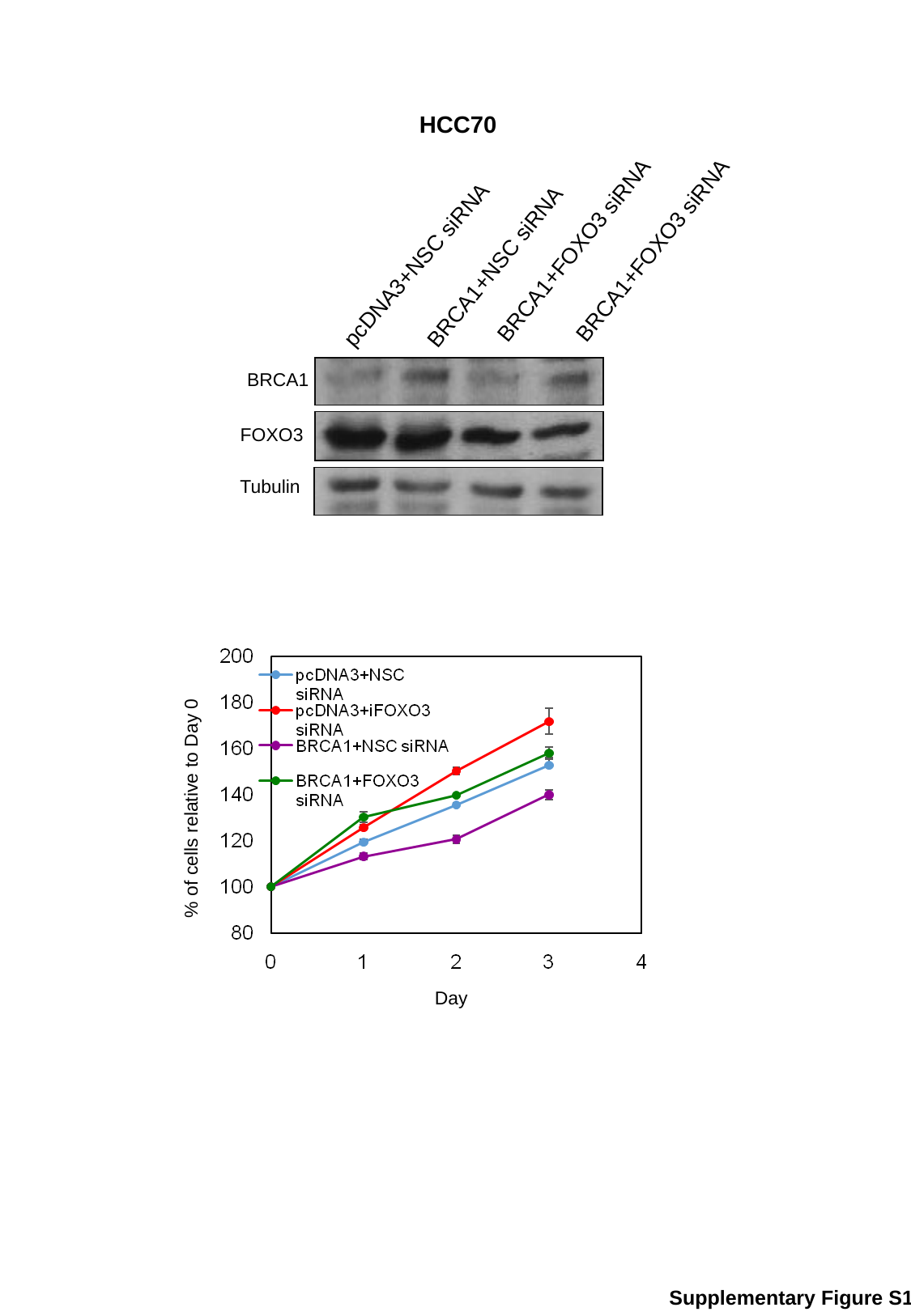

HCC70
BRCA1+FOXO3 siRNA
BRCA1+FOXO3 siRNA
pcDNA3+NSC siRNA
BRCA1+NSC siRNA
BRCA1
FOXO3
Tubulin
% of cells relative to Day 0
Day
Supplementary Figure S1
