# Supplementary material for: BRCA1 positively regulates FOXO3 expression by restricting FOXO3 gene methylation and epigenetic silencing through targeting EZH2 in breast cancer
Source: Oncogenesis. 2016 Apr 4;5(4):e214–. doi: 10.1038/oncsis.2016.23 (PMC4848836; doi:10.1038/oncsis.2016.23)
Supplement: Supplementary Figure 2 [file oncsis201623x4.ppt]

## Slide 1
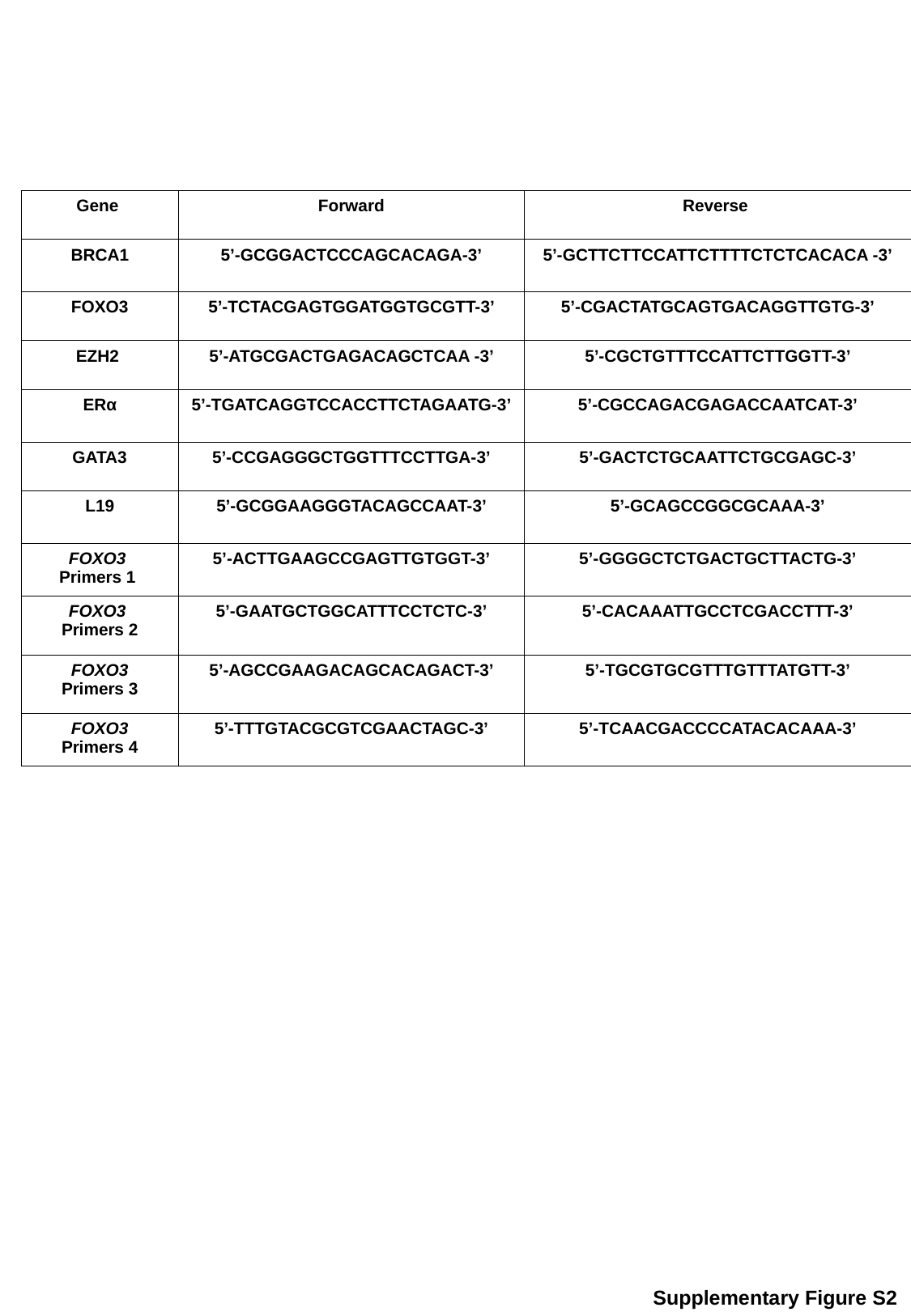

| Gene | Forward | Reverse |
| --- | --- | --- |
| BRCA1 | 5’-GCGGACTCCCAGCACAGA-3’ | 5’-GCTTCTTCCATTCTTTTCTCTCACACA -3’ |
| FOXO3 | 5’-TCTACGAGTGGATGGTGCGTT-3’ | 5’-CGACTATGCAGTGACAGGTTGTG-3’ |
| EZH2 | 5’-ATGCGACTGAGACAGCTCAA -3’ | 5’-CGCTGTTTCCATTCTTGGTT-3’ |
| ERα | 5’-TGATCAGGTCCACCTTCTAGAATG-3’ | 5’-CGCCAGACGAGACCAATCAT-3’ |
| GATA3 | 5’-CCGAGGGCTGGTTTCCTTGA-3’ | 5’-GACTCTGCAATTCTGCGAGC-3’ |
| L19 | 5’-GCGGAAGGGTACAGCCAAT-3’ | 5’-GCAGCCGGCGCAAA-3’ |
| FOXO3 Primers 1 | 5’-ACTTGAAGCCGAGTTGTGGT-3’ | 5’-GGGGCTCTGACTGCTTACTG-3’ |
| FOXO3 Primers 2 | 5’-GAATGCTGGCATTTCCTCTC-3’ | 5’-CACAAATTGCCTCGACCTTT-3’ |
| FOXO3 Primers 3 | 5’-AGCCGAAGACAGCACAGACT-3’ | 5’-TGCGTGCGTTTGTTTATGTT-3’ |
| FOXO3 Primers 4 | 5’-TTTGTACGCGTCGAACTAGC-3’ | 5’-TCAACGACCCCATACACAAA-3’ |
Supplementary Figure S2
